# Autoantibodies Against the Node of Ranvier in Seropositive Chronic Inflammatory Demyelinating Polyneuropathy: Diagnostic, Pathogenic, and Therapeutic Relevance

**DOI:** 10.3389/fimmu.2018.01029

**Published:** 2018-05-14

**Authors:** Atay Vural, Kathrin Doppler, Edgar Meinl

**Affiliations:** ^1^Institute of Clinical Neuroimmunology, Biomedical Center, University Hospitals, Ludwig-Maximilians-Universität München, Planegg-Martinsried, Germany; ^2^Research Center for Translational Medicine, Koç University, Istanbul, Turkey; ^3^Department of Neurology, University Hospital Würzburg, Würzburg, Germany

**Keywords:** autoantibody, seropositive, chronic inflammatory demyelinating polyneuropathy, node of Ranvier, paranode, neurofascin, contactin, contactin-associated protein 1

## Abstract

Discovery of disease-associated autoantibodies has transformed the clinical management of a variety of neurological disorders. Detection of autoantibodies aids diagnosis and allows patient stratification resulting in treatment optimization. In the last years, a set of autoantibodies against proteins located at the node of Ranvier has been identified in patients with chronic inflammatory demyelinating polyneuropathy (CIDP). These antibodies target neurofascin, contactin1, or contactin-associated protein 1, and we propose to name CIDP patients with these antibodies collectively as seropositive. They have unique clinical characteristics that differ from seronegative CIDP. Moreover, there is compelling evidence that autoantibodies are relevant for the pathogenesis. In this article, we review the current knowledge on the characteristics of autoantibodies against the node of Ranvier proteins and their clinical relevance in CIDP. We start with a description of the structure of the node of Ranvier followed by a summary of assays used to identify seropositive patients; and then, we describe clinical features and characteristics linked to seropositivity. We review knowledge on the role of these autoantibodies for the pathogenesis with relevance for the emerging concept of nodopathy/paranodopathy and summarize the treatment implications.

## Key Points

– Autoantibodies against neurofascin, contactin1, or contactin-associated protein 1 (Caspr) occur in approximately 10% of chronic inflammatory demyelinating polyneuropathy (CIDP) patients. We propose to call these collectively seropositive.
– These autoantibodies target nodal and paranodal structures and typically have an IgG4 isotype.
– Unlike seronegative CIDP, there is no overt inflammation and demyelination in these patients.
– The pathology caused by these antibodies is named as nodopathy/paranodopathy, which is characterized by dissection of myelin loops from axon at the paranode and subsequent axonal degeneration.
– Seropositive CIDP patients have a specific clinical phenotype that is distinct from seronegative CIDP. They typically respond poorly to IVIg but may benefit from plasmapheresis and rituximab (RTX).
– Thus, these autoantibodies have a diagnostic and prognostic value as a biomarker in CIDP.
– Antibodies to neurofascin155 have also been reported in some patients with combined central nervous system (CNS) and peripheral nervous system (PNS) inflammation; however, the clinical relevance of this finding is not known at the moment.

## Introduction

The molecular composition of the node of Ranvier has been characterized in the last 20 years (1–4), and autoantibodies that target these proteins, namely neurofascin, contactin1, and Caspr have been identified in chronic inflammatory demyelinating polyneuropathy (CIDP) in the past 6 years (5, 6). The presence of these autoantibodies has been shown consistently in several studies and, although they are present in only about 10% of the patients with CIDP, their presence has been linked to different clinical and prognostic features compared to the patients who lack these antibodies. Therefore, we grouped the subset of patients with antibodies against neurofascin, contactin1, and Caspr under the term “seropositive CIDP.” While antibodies to gangliosides are frequently found in GBS, such autoantibodies are typically not found in CIDP (6). In this article, we

(1) introduce the molecular components of the node of Ranvier
(2) summarize the clinical phenotype of seropositive CIDP patients
(3) review the treatment approaches
(4) discuss the evidence for their pathogenic relevance and the concept of nodopathy
(5) summarize the diagnostic methods to identify these seropositive patients.

## Structure of the Node of Ranvier

Segregation of the voltage-gated sodium channels in axonal domains instead of diffusely floating on the membrane is a critical step during the evolution of vertebrates (7). This was followed by the myelination of the axons in higher vertebrates. These improvements provided organisms with a faster and more energy efficient way of conducting electrical signals throughout the longer and thicker axons, which is called saltatory propagation. Action potentials are generated in the axon initial segments (AIS) and regenerated in each node of Ranvier. AIS and nodes are also important for the adjustment of conduction velocity of individual axons to achieve synchronization at the network level (8). Thus, nodes of Ranvier are the critical components of the myelinated axons and they are not just uniform, passive sites where sodium channels are concentrated.

### General Organization of the Axon

In unmyelinated axons, sodium channels are found diffusely throughout the axon. In myelinated axons, these channels are concentrated in the AIS and the nodes by means of their anchoring motifs and the scaffold proteins (9). The scaffold of the axon is an orderly organized structure in the AIS and the nodes of Ranvier (10, 11). Circular actin bands are spaced every 190 nm perpendicular to the axolemma. Spectrins bind to actin circles and are located longitudinally just below the axonal membrane to provide an anchoring platform for the extracellular ion channels and cell adhesion proteins. Ankyrins mediate the binding of membrane-bound proteins with spectrins (12). The axonal domain located between the two nodes is called the internode. The length of the internode can reach 1 mm or more whereas the nodes have only a length of 1 µm (2).

**Figure 1:**
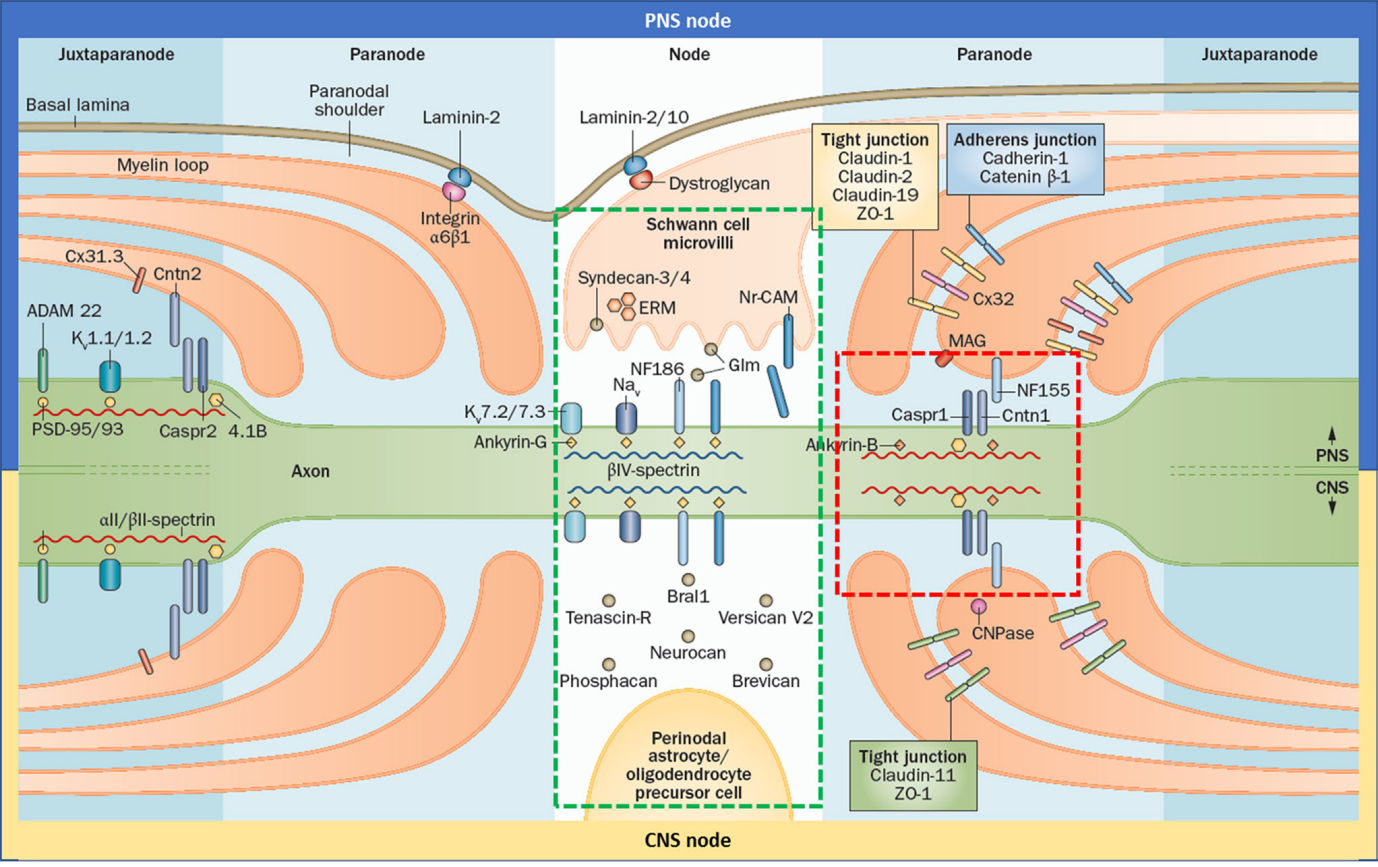
Gross and molecular structure of the node of Ranvier in central nervous system (CNS) and peripheral nervous system (PNS). Cell adhesion molecules forms complex with other adhesion molecules, ion channels, and the cytoskeleton to form the nodal, paranodal, and juxtapranodal compartments. The main difference between the CNS and PNS nodes is the interaction partners of neurofascin 186 (NF186). The targets of the autoantibodies found in patients with seropositive chronic inflammatory demyelinating polyneuropathy are neurofascin 155, Cntn1, and contactin-associated protein 1, which altogether form a complex in the paranode (red rectangle); and NF186 that is located in the node (green rectangle) and also in the axon initial segment. Reprinted by permission from Springer Customer Service Centre GmbH: Springer Nature [COPYRIGHT] (5).

The node of Ranvier is composed of three subdomains: node, paranode, and juxtaparanode (JXP) (Figure 1). Cell adhesion molecules, cytoskeletal elements, and extracellular matrix proteins all contribute to the formation of these subdomains. In the literature, the term “node” is used for both the complex that is composed of all three subdomains and also to refer only to the nodal subdomain. Throughout this review, we use “the node of Ranvier” to refer to the whole complex and “node” to refer to the specific subdomain.

### Node and AIS

Nodes are very active sites. Although they occupy only 0.1–0.3% of axonal surface, nodes contain 90% of the organelles that are found in the whole length of the axon and there is an active vesicle trafficking in the nodes (13). Axon thickness is diminished in the nodal and paranodal domains. Thus, nodes present a bottleneck for the anterograde and retrograde axonal transportation (14).

Neurofascin 186 (NF186) is the cell adhesion molecule that anchors voltage-gated sodium channels in the node and AIS (Figure 1). It is a transmembrane protein with six immunoglobulin (Ig)-like domains, four fibronectin type III (Fn) domains, and one mucin domain (15). Its binding partner is ankyrin G inside the cell which in turn interacts with βIV-spectrin (16). In the PNS, NF186 interacts extracellularly with the soluble gliomedin and NrCAM that is located on the Schwann cell microvilli that fills the nodal space whereas in the CNS, NF186 interacts with extracellular matrix proteins.

The axonal initial segment is a specialized ~30 nm domain that is located next to the soma (12). The AIS contains a high number of ion channels and it is responsible for the initial formation of action potentials. Many of the structural components of AIS, including sodium and potassium channels, NF186, NrCAM, and their binding partners in the cytoskeleton, are identical to the node (17). Thus, autoantibodies against these elements would probably also affect the AIS.

### Paranode

Paranodal junction (PNJ) is by far the largest intercellular junction known (18). Size of the PNJ depends on the number of myelin lamella. An axon with 50 paranodal myelin loops has a 5 µm long paranode, much longer than the node (19). The largest axons may have up to 250–300 turns of myelin (20). PNJ is formed by the regularly spaced heterotrimeric complexes of contactin1 (CNTN1) and contactin-associated protein 1 (Caspr) on the axonal side, and neurofascin 155 (NF155) on the glial side (Figure 1) (21). These junctional complexes are also named as transverse bands because of their appearance under electron microscopy (22).

Neurofascin 155 is the glial isoform of NF186 which lacks its mucin domain and has an Fn3 domain instead of an Fn5 domain (23). It is alternatively spliced in the myelin-producing glia by an RNA-binding protein named Quaking (24). NF155 interacts with CNTN1 through its Ig domains (25). The cytoplasmic domain of the NF155 interacts with the ankyrin proteins (26). It is a glycoprotein like other cell-adhesion proteins and has eight glycosylation sites. Glycans of NF155 participate in the formation of complexes and are also important for autoantibody binding (27, 28). NF155 is palmitoylated and is located on the sulfatide-rich lipid rafts (29, 30). Integrity of the PNJ is maintained not only through protein–protein interactions but also by virtue of the lipid rafts. Transgenic mice that are incapable of synthesizing sulfatides show a severe phenotype similar to neurofascin knock-out mice (31).

Contactin1 is a glycosylphosphatidylinositol-anchored protein. It has six Ig domains and four Fn domains, similar to neurofascin and it forms a complex with Caspr on the axonal side (3). The interaction of these two proteins is critical for their trafficking to the paranode (32). Caspr binds to CNTN1 in the endoplasmic reticulum and together they bypass the conventional protein trafficking pathway that pass through Golgi and the complex is sent directly to the paranode. In this situation, CNTN1 carries a lower molecular weight mannose-rich glycan. This type of glycan side chain is critical for its binding with NF155. In the absence of Caspr, CNTN1 is sent to the node instead of the paranode, after modification with complex glycans in the Golgi apparatus (32). Caspr is a transmembrane glycoprotein which has laminin G-like domains, EGF-like cysteine-rich domains, a PGY motif, an amino-terminal discoidin domain and a 4.1 binding domain (33, 34). Intracellularly, it is bound to protein 4.1B, and α2/β2 spectrins (35).

The main function of the PNJ is to block the passage of nodal currents into the internode. This is crucial for the saltatory conduction and its loss may result in conduction block (36). However, PNJs are not completely impermeable to all molecules. Studies with different molecular weight dextrans showed that molecules as large as 160 Å (almost the size of an IgG) can pass through the PNJ (37). However, this does not happen through the 3 nm space between the axonal and glial membranes or the narrow channel between the junctional complexes. Instead, there is a triangle-shaped larger but much longer (100×) pathway between the adjacent myelin loops and axolemma. This long channel does not permit the diffusion of ions which are removed quickly from the nodal space but allows the slow diffusion of nutrients and larger molecules (37).

The PNJ is also directly involved in the formation and maintenance of the node of Ranvier. During formation, PNJ restricts nodal proteins to the node and thereafter it acts as a diffusion barrier for the movement of voltage-gated ion channels found in the node and the JXP (4). Developmental knock-out mouse models of the PNJ complex proteins show a severe phenotype with severe ataxia, hind limb paresis, and death before the third postnatal week (36, 38, 39). Electrophysiological studies reveal dramatic reductions in the nerve conduction velocities. When NF155 was ablated during adulthood by knock-down of QKI, mice progressively developed hind limb paralysis with visible thoracic kyphosis and numerous mice reached a clinical endpoint of accelerated breathing and immobility (24). Interestingly, these mice also displayed a characteristic shaking phenotype, reminiscent of the tremor seen in the CIDP patients with anti-NF155 antibodies.

### Juxtaparanode

In the JXP, Caspr2 binds to contactin2 on the axonal side and interacts in trans with the glial contactin2 that is expressed on the myelinating glia (Figure 1). Caspr2 has a similar structure as Caspr, showing 45% identity at the amino acid level (40). However, it lacks the PGY motif, which plays an important role in trafficking of CNTN1 to the paranode, and has an additional PDZ binding domain. Intracellularly, Caspr2 binds to the protein 4.1 that plays an important role in the association of Caspr2 with Kv1 channels and their clustering at the JXP (41). The AIS also contains several JXP components including Kv1 channels and Caspr2. Despite this molecular similarity, there are fundamental differences in the mechanisms that control ion channel distribution at the nodes of Ranvier and the AIS (4). At the JXP, clustering of Kv1 channels requires axon–glia interaction mediated by the Caspr2/Contactin2 adhesion complex, but not PSD proteins. In contrast, clustering of Kv1 channels at the AIS depends on the presence of PSD-93 but not on Caspr2. Besides, Caspr2 is found commonly also in inhibitory interneurons in the hippocampus, which explains the encephalopathy seen in the majority of patients with anti-Caspr2 autoantibodies (42). These autoantibodies also cause hyperexcitable peripheral nerves in many patients, however whether this is due to the dysfunction of the JXP or AIS is not exactly known at the moment. As the JXP is sequestered behind the paranode, autoantibodies may not be able to reach that site easily.

### Differences Between the PNS and CNS Node

Although the overall structure and elements of the PNS and CNS are similar, some differences exist (Figure 1). In PNS, the nodal space is filled with the microvilli that originate from the Schwann cells. In PNS nodes, Schwann cells secrete gliomedin to the nodal space which form trimers. These trimers bind to NrCAM that is present on the microvillar membrane. This complex creates a high avidity site for NF186-binding, promoting their aggregation to the node (43). In CNS, oligodendrocytes do not form microvilli and the nodal space is filled with extracellular matrix proteins instead. Here, NF186 interacts with the extracellular matrix proteins including versican, brevican, and others (4). In terms of the paranode, the only difference identified so far is that NF155 interacts with ankyrin G in the CNS and ankyrin B in PNS (26). There are also differences in the tight junctions (Figure 1).

## Methods to Identify Targets of Nodal Autoantibodies

Neurofascin has been identified as a target of autoantibodies with a proteomic approach (44): Glycoproteins purified from human myelin by lentil-lectin affinity chromatography were separated by two-dimensional gel electrophoresis and blotted. The spots that were recognized by IgG of an MS patient were analyzed by mass-spectroscopy and this yielded neurofascin (44). Subsequently, human NF155 and NF186 were produced recombinantly for an enzyme-linked immunosorbent assay (ELISA), cell-based assays (CBA) with human NF155 and NF186 were established, and anti-NF155 Abs were found in a proportion of patients with CIDP by using these assays (27).

CNTN1 and Caspr were identified by using an approach that combines tissue-based assays (TBA), proteomics, and CBA (45, 46). Incubation of the patients’ sera with rodent-teased fibers showed colocalization of autoreactive antibodies with the nodal antigens in several patients. Subsequently, the target antigen of the reactive antibodies in patients’ sera were identified by immunoprecipitation of the antigen–antibody complexes after incubating patients’ sera with neuronal culture cells, followed by gel separation and analysis with mass spectrometry (45). Presence of NF155 and NF186 autoantibodies was verified independently by using a similar approach (28, 47).

## Methods to Detect Autoantibodies Against Nodal Proteins in Patient Cohorts

CIDP-related autoantibodies so far identified react with cell surface proteins that are found in their native three or four dimensional form. TBA provide an efficient method to screen the presence of such antibodies. In this assay, binding of antibodies in patient sera to rodent brain, spinal cord, or peripheral nerve tissue sections is determined. When reactivity is detected, specific tests are necessary to identify the target antigen of these autoantibodies (Table 1).

**Table 1:** Methodologies and disease groups of the previous studies testing antibodies against nodal proteins in chronic inflammatory demyelinating polyneuropathy (CIDP) and peripheral neuropathies.

	Assay method						
Study (year)	ELISA species of the antigen	CBA species of the antigen	Rodent nerve IF	Rodent brain/spinal cord IHC	Targets of antibodies tested	Patient Groups	Reference
Prüss et al. (2011)	Rat	–	–	–	Neurofascin, CNTN2	GBS	(48)
Ng et al. (2012)	Human	Human	–	+	NF155, NF186	CIDP, GBS	(27)
Querol et al. (2013)	–	Human	+	–	CNTN1	CIDP, GBS	(45)
Kawamura et al. (2013)	Rat	Human	+	+	NF155	CCDP	(49)
Querol et al. (2014)	Human	Human	+	+	NF155, NF186	CIDP, GBS	(50)
Notturno et al. (2014)	Rat peptide	Human	+	–	NF186, gliomedin	MMN	(51)
Ogata et al. (2015)	–	Human	+	–	NF155, NF186	CIDP, GBS, MS	(52)
Vural et al. (2015)	Human	–	–	–	NF155	CCPD	(53)
Doppler et al. (2015a)	Human	Rat	+	+	CNTN1	CIDP, GBS	(54)
Doppler et al. (2015b)	Human	Human & Rat	+	–	NF155, NF186, CNTN1	MMN	(55)
Miura et al. (2015)	Human	Human	+	+	CNTN1	CIDP, GBS, MS	(56)
Devaux et al. (2016)	Human	Human	+	+	NF155, NF186	CIDP, GBS, MS	(28)
Cortese et al. (2016)	Human	Human	+	–	NF155	CCPD, CIDP, MS	(57)
Doppler et al. (2016)	–	Human	+	+	Caspr	CIDP, GBS	(46)
Kadoya et al. (2016)	Human	Human	+	–	NF155	CIDP, GBS, MS	(58)
Mathey et al. (2017)	Human	Human	+	+	NF155, NF186, CNTN1, gliomedin	CIDP, MMN	(59)
Delmont et al. (2017)	Human	Human	+	+	NF186, NF140, gliomedin, NF155, CNTN1, Caspr	CIDP, GBS, MS	(47)
Querol et al. (2017)	Human	Human	+	–	NF155, CNTN1, NrCAM, gliomedin, CNTN1/Caspr & CNTN2/Caspr2 complexes, NavB1 and NavB2, gangliosides, MPZ, PMP2	CIDP	(6)
Koike et al. (2017)^a^	Human	Human	Human	–	NF155, CNTN1	CIDP	(60)
Garg et al. (2017)	Human	–	–	–	NF155	CIDP	(61)
Burnor et al. (2018)	–	Mouse & Rat	–	–	NF155, NF186, NF140	CIDP, GBS, genetic neuropathy, idiopathic neuropathy	(62)

*^a^Results of the anti-NF155 antibody testing were reported in Ref. (58)*.

*ELISA, enzyme-linked immunosorbent assay; CBA, cell-based assay; IF, immunofluorescence; IHC, immunohistochemistry; CNTN2, contactin2; GBS, Guillain-Barre Syndrome; NF155, neurofascin 155; NF186, neurofascin 186; CIDP, chronic inflammatory demyelinating polyneuropathy; CNTN1, contactin1; CCPD, combined central and peripheral demyelination; MMN, multifocal motor neuronopathy; MS, multiple sclerosis; Caspr, contactin-associated protein 1; NF140, neurofascin 140; NrCAM, neuronal cell adhesion molecule; MPZ, myelin protein zero; PMP2, peripheral myelin protein 2*.

In earlier studies, peptide-based ELISA and western-blotting were used for that purpose, but they were found to have a low specificity. This is intuitive as these assays utilize peptide fragments or denatured proteins instead of native proteins. Recent development of CBA provided an efficient method to detect autoantibodies against epitopes that are found in the native form of proteins (Table 1). In CBA, mammalian culture cells transfected with the specific target protein are incubated with patient serum. Another sensitive method is ELISA (Table 1). The expression system used to synthesize target protein for ELISA is also important as proteins expressed by non-human cells may have different post-translational modifications, which in turn may affect the antibody–antigen interaction. In a previous study, only a few serum samples from CIDP patients showed reactivity to NF155 and NF186 expressed in HEK293-EBNA cells, whereas virtually all donors showed some response against ratNF155 derived from NS0 murine myeloma cells (27).

The utility of CBA or ELISA to detect autoantibodies differs between different antigens. It may be difficult to interpret the results in some patients despite the usage of a specific assay if the result is near the cut-off value. Test results should be evaluated together with the clinical picture of the patient and repeated testing of serum taken during different phases of the disease may provide valuable information.

## Clinical Implications of Antibodies Against Nodal Antigens

### Nodal Autoantibodies

Neurofascin 186 and gliomedin have been the usual suspects since the node of Ranvier antigens came into focus as targets of autoantibodies in GBS and CIDP (63). However, several consecutive studies that used native human gliomedin could not identify its presence in these disorders, and NF186 could only be detected in two studies indicating their rarity (27, 47) (Table 2).

**Table 2:** Studies that tested autoantibodies against neurofascin 186 (NF186) and gliomedin by using native human proteins.

	NF186			Gliomedin				
Study (year)	CIDP	GBS	MMN	CIDP	GBS	MMN	Isotype	Reference
Ng et al. (2012)	0/119	2/115	–	–	–	–	IgG1, IgG3	(27)
Querol et al. (2014)	0/53	0/51	0/22	–	–	–	–	(50)
Ogata et al. (2015)	0/50	0/26	–	–	–	–	–	(52)
Doppler et al. (2015b)	–	–	0/33^a^	–	–	–	–	(55)
Devaux et al. (2016)	0/533	0/200	–	–	–	–	–	(28)
Mathey et al. (2017)	0/44	–	0/15^a^	0/44	–	0/15	–	(59)
Delmont et al. (2017)	5/246	0/26	–	0/246	0/26	–	IgG4, IgG3	(47)
Burnor et al. (2018)	1/40^b^	0/14	–	–	–	–	IgG4	(62)

**Total**	**6/1046**	**2/432**	**0/70**	**0/290**	**0/26**	**0/15**		

*^a^NF155 also negative*.

*^b^Serum from this patient was reactive to NF186, NF155, and NF140*.

*NF186, neurofascin 186; CIDP, chronic inflammatory demyelinating polyneuropathy; GBS, Guillain-Barre Syndrome; MMN, multifocal motor neuronopathy*.

In a large European cohort of 246 CIDP patients, 5/246 patients had anti-NF186 antibodies (47). These antibodies had a similar reactivity to NF140, which is predominantly expressed at early developmental stages and also strongly expressed in lesions of MS patients (64), and NF155. Sera from these patients stained both the nodes and AIS at the same time (47). Compatible with this finding, the patients had a more severe and subacute onset compared to antibody-negative CIDP patients. Sensory ataxia was common and average age of onset was older. Electrophysiologically, there was conduction block that reversed after immunotherapy. Importantly, intravenous immunoglobulin (IVIG) response was better (47) compared to anti-NF155 positive patients (28, 50, 52, 58). In a separate study, a 50-year-old man with a very severe form of CIDP, who almost progressed into a locked-in state, had antibodies that recognize all three neurofascin isoforms, with higher anti-NF186 titers compared to anti-NF155 (62).

Antibodies against NF186 have also been analyzed in MMN in several studies; however, none of the patients was found to be positive by testing against native human proteins so far (50, 55, 59) (Table 2). In only one study, 60% of the patients with MMN had antibodies against anti-NF186 and anti-gliomedin; however, this study is methodologically different from others as rat peptides were used for detection of autoantibodies (51).

### Paranodal Autoantibodies

#### Anti-NF155

##### Chronic Inflammatory Demyelinating Polyneuropathy

Anti-NF155 antibodies have been consistently reported in a fraction of patients with CIDP (Table 3) and have been strongly associated with HLA-DRB1*15 (65). The reported frequency is between 4 and 18% and it was 7% in a large cohort of 533 patients (27, 28, 50, 52, 58, 59, 61). In several studies, a specific clinical phenotype that differs from the antibody negative CIDP has been described (Table 3). This includes a younger age of onset around 20–30 years instead of 50–60, a subacute and more severe onset, disabling tremor, sensory and cerebellar ataxia, distal dominant weakness, and poor response to IVIG (28, 50, 52, 58). Laboratory and electrophysiological findings that were associated with anti-NF155 antibodies were higher levels of cerebrospinal fluid protein and marked prolongation of distal and F-wave latencies (52, 58). Additionally, gadolinium enhancement and enlargement in the spinal roots and plexuses have been described in patients undergoing magnetic resonance (MR) neurography (52). Diffuse peripheral nerve enlargement and cranial nerve hypertrophy have also been reported (61, 66).

**Table 3:** Studies that tested autoantibodies against neurofascin 155 (NF155) by using native human proteins.

	Diagnosis						
Study (year)	CIDP	GBS	CCPD	MS	Isotype	Clinical characteristics	Reference
Ng et al. (2012)	6/119	2/115	–	–	IgG4 (CIDP) IgG1(GBS)	Positive response to plasma exchange	(27)

Kawamura et al. (2013)	0/16	0/20	5/7	0/20	Not done	Peripheral nerve demyelination is indistinguishable from CIDP, CNS involvement is mostly typical for MS, CSF OCB are negative mostly, response to corticosteroids is limited	(49)

Querol et al. (2014)	2/53 and 2/8^a^	0/51	–	–	IgG4	Severe phenotype, poor response to IVIG, and disabling tremor	(50)

Ogata et al. (2015)	9/50	1/26	–	0/32	IgG4 (CIDP) IgG1(GBS)	Younger onset age, tremor, extremely high CSF protein levels, symmetric spinal root and plexus hypertrophy, and marked prolongation of distal and F-wave latencies	(52)

Vural et al. (2015)	–	–	0/5	–	–	–	(53)

Devaux et al. (2016)	38/533	0/200	–	0/100	IgG4	Younger age at onset, subacute onset, sensory and cerebellar ataxia, tremor, CNS demyelination, poor response to IVIG	(28)

Cortese et al. (2016)	1/26	–	0/16	0/15	IgG4	Distally predominant weakness, ataxia, tremor, and IVIG resistance	(57)

Kadoya et al. (2016)	15/191	0/57	–	0/16	IgG4	Younger CIDP onset, distal dominant phenotype, tremor, and sensory ataxia, higher levels of CSF protein, poor response to IVIG, mRS scores at diagnosis was higher, patients underwent PE more frequently	(58)

Mathey et al. (2017)	3/44	–	–	–	IgG4	–	(59)

Delmont et al. (2017)	9/246	0/26	–	0/52	–	–	(47)

Garg et al. (2017)	3/55	–	–	–	–	Sensory ataxia, tremor (in 1/3), diffuse nerve enlargement, IVIG resistance	(61)

Burnor et al. (2018)	4/40	1/14	–	–	IgG4 (CIDP) IgM (GBS)	High CSF protein; severe, progressive CIDP; poor response to IVIG	(62)

**Total**	**90/1403**	**4/496**	**5/28**	**0/235**			

*^a^Among IVIG resistant CIDP*.

*CIDP, chronic inflammatory demyelinating polyneuropathy; GBS, Guillain-Barre Syndrome; CCPD, combined central and peripheral demyelination; MS, multiple sclerosis; CNS, central nervous system; CSF, cerebrospinal fluid; OCB, oligoclonal bands; IVIG, intravenous immunoglobulin; mRS, modified Rankin score; PE, plasma exchange*.

Although early studies reported that anti-NF antibodies are associated with GBS, succeeding studies performed with native human antigens produced in mammalian cell lines showed that these antibodies are only very rarely (<1%) positive in patients with GBS (63, 48).

##### Combined Central and Peripheral Demyelination (CCPD)

Anti-NF155 antibodies have also been described in CCPD patients from Japan in 5/7 CCPD patients (49). The researchers used ELISA with native rat NF155 as the initial screening method and confirmed the results with a cell based assay that expressed human NF155. Most of the anti-NF155 positive CCPD patients in this study had typical findings of both MS and CIDP. However, oligoclonal bands were negative in 4/5 patients and response to corticosteroids was only partially effective in most patients. In an additional study again from Japan, anti-NF155 antibodies were reported in 5/11 of the CCPD patients tested, but information regarding methodology of testing and clinical characteristics of these patients were not present in this article (67). Another study investigated the presence of CNS demyelination in CIDP patients that are positive for anti-NF155 antibodies and found that 3/38 of these patients had additional signs of central demyelination, compared to none in antibody-negative CIDP patients (28). Signs of central demyelination in these patients were much less prominent compared to the patients reported by Kawamura et al. (49). In this study, sera from anti-NF155 antibody positive CIDP patients were also incubated with rat brain tissues and reactivity were positive regardless of the presence or absence of central demyelination in patients.

Two studies tested CCPD patients of non-Japanese origin for the presence of anti-NF155 antibodies and none of the patients were positive (Table 3) (53, 57). In the first study (53), four patients who fulfilled the criteria for both MS and CIDP with a positive response to plasmapheresis were tested by ELISA using human NF155 and NF186. Interestingly, three of these patients had clinical characteristics of anti-NF155 antibody-positive CIDP including young age of onset, a subacute and severe onset of CIDP, lack of response to IVIG, diffusely enlarged, gadolinium enhancing nerve roots, and high CSF protein levels. One patient had also disabling tremor. These findings suggest that other antibodies may also be involved in CCPD patients (53). In the second study, patient cohort included 16 patients, three of whom presented as young-onset MS plus CIDP syndrome similar to the Japanese cohort, but they were seronegative. Similarly, 13 patients who presented as older-onset myeloneuroradiculitis with or without encephalopathy in the same cohort also were seronegative for anti-NF155 antibodies (57). Recently, an NF-155- and NF-186-specific T cell response without Abs to NF was described in a patient developing pontocerebellar demyelination after 10 years of CIDP (68).

Possible reasons for the different abundance of anti-NF155 in CCPD in the published studies include differences in ethnicity [Caucasian (53, 57) vs Japanese (49, 67)] and also the heterogeneity of CCPD.

#### Anti-CNTN1

Contactin1, the binding partner of NF155 on the neuronal side, is another target of autoantibodies in CIDP. Anti-CNTN1 antibodies were first described by Querol et al. (45) and then further characterized by subsequent studies (47, 54, 56, 59, 60) (Table 4). Frequency of these antibodies in CIDP patients is between 3 and 8%. Anti-CNTN1 antibodies are associated with specific clinical features including a more advanced age of onset compared to antibody-negative CIDP, an aggressive and GBS-like subacute onset of weakness, a very high ratio of sensory ataxia, early axonal involvement and poor response to IVIG. Tremor may also be more common in these patients than antibody-negative patients, despite being less frequent compared to anti-NF155 positive patients. Corticosteroids were effective. These antibodies were also tested in patients with GBS and MMN, but none were positive (Table 4).

**Table 4:** Studies that tested autoantibodies against CNTN1 by using native human proteins.

	Diagnosis					
Study (year)	CIDP	GBS	MMN	Isotype	Clinical characteristics	Reference
Querol et al. (2013)	3/46	0/48	–	IgG4	Advanced age, predominantly motor involvement, aggressive symptom onset, early axonal involvement, poor response to IVIG	(45)

Miura et al. (2015)	13/533	0/200^a^	–	IgG4	Subacute onset of symptoms, sensory ataxia, poor response to IVIG, good response to corticosteroids	(56)

Doppler et al. (2015a)	4/53	0/51	–	IgG4 & IgG3	Acute onset of disease, severe motor symptoms, tremor, partial response to IVIG	(54)

Doppler et al. (2015b)	–	–	0/33	–	–	(55)

Mathey et al. (2017)	3/44	–	0/15	IgG4	–	(59)

Delmont et al. (2017)	2/246	0/26	–	–	–	(47)

Koike et al. (2017)	1/131	–	–	IgG4	–	(60)

**Total**	**26/807**	**0/325**	**0/48**			

*^a^5 pts with GBS had IgG2 abs but did not stain-teased fibers*.

*CIDP, chronic inflammatory demyelinating polyneuropathy; GBS, Guillain-Barre Syndrome; MMN, multifocal motor neuronopathy; IVIG, intravenous immunoglobulin*.

#### Anti-Caspr

Initially, anti-Caspr antibodies have been found in one patient with CIDP and another patient with GBS, both of whom had very severe pain that necessitated treatment with high dose pregabalin and opioids as a distinguishing feature (Table 5) (46). Sera from these patients reacted with small TRPV1 positive neurons in the dorsal root ganglia, potentially explaining the cause of severe pain. Apart from this finding, clinical features were quite similar to the patients with antibodies against the other paranodal antigens. The patient diagnosed with CIDP was young (30 years old) and onset of disease was subacute, severe, and motor dominant. He was unresponsive to IVIG and methylprednisolone and required walking-aid in a few months. Remarkably, pain resolved quickly after effective immunotherapy in both patients. In the patient with CIDP, response to RTX was very good and the patient could walk independently after 20 months of therapy. Two more patients have been reported recently, validating the rare presence of these autoantibodies in CIDP (47).

**Table 5:** Studies that tested autoantibodies against contactin-associated protein 1.

	Diagnosis				
Study, year	CIDP	GBS	MS	Isotype	Clinical characteristics
Doppler et al. (2016)	1/35	1/22	–	IgG4 (CIDP) and IgG3 (GBS)	Severe pain; subacute, severe, motor dominant onset, reversible conduction block, unresponsive to IVIG, and corticosteroids

Delmont et al. (2017)	2/246	0/26	0/52	–	–

**Total**	**3/281**	**1/48**	**0/52**		

*CIDP, chronic inflammatory demyelinating polyneuropathy; GBS, Guillain-Barre Syndrome; MS, multiple sclerosis; IVIG, intravenous immunoglobulin*.

### Summary of the Clinical Relevance of Antibodies Against the Node of Ranvier

The data reviewed in this paper show that CIDP is associated with antibodies mainly against paranodal proteins (NF155, CNTN1, and rarely Caspr) and to a lesser extent NF186 in around 10% of patients. These patients designated seropositive CIDP have different clinical features compared to seronegative CIDP (28, 59) (Tables 2–5). While there is now consensus that a proportion of patients with CIDP has autoantibodies against NF, Caspr, or CNTN1, the presence of autoantibodies against the myelin proteins such P2, P0, PMP-22, and connexin has been described in some reports, but this was not confirmed by others (69).

Anti-NF155 antibodies may also be associated with CCPD, but this topic requires further studies. Despite early reports, anti-gliomedin antibodies directed against the conformational epitopes could not be shown in patients with inflammatory demyelinating disorders, yet. In GBS, antibodies against NF155, NF186, and Caspr may be detected only very rarely (<1%) suggesting their limited usage as a biomarker in this disorder (Tables 2, 3 and 5). Instead, the detection of these antibodies in GBS patients may be a marker for the development of acute-onset CIDP. In MMN, autoantibodies against NF186, Gliomedin, and CNTN1 are typically not found (Tables 2–5).

## Ig Isotypes

The dominant isotype of the autoantibodies in seropositive CIDP patients is IgG4 (Tables 2–5). All reported patients (90/90) with anti-NF155, 22/24 of anti-CNTN1 positive patients, one patient with anti-Caspr antibody, and 5/6 of anti-NF186 positive patients had IgG4. Only two patients with anti-CNTN1 and one patient with anti-NF186 antibodies had IgG3 as the dominant subclass (47, 54). Anti-CNTN1 antibodies were of IgG2 isotype in three patients; however, these sera did not react with murine nerve fibers and not considered as positive or pathogenic. Of note, complement-fixing IgG1 (27, 52, 56, 59, 62), IgG2 (47, 50, 52, 54, 56, 62, 70), and IgG3 subtype antibodies (71) were also detected in the sera of some CIDP patients concomitant with but at lower levels than IgG4.

IgG4 has several characteristic features different from the other IgG subtypes (Box 1) (72, 73). IgG4 represents approximately 5% of the total IgG pool. Repeated or long-term exposure to non-infectious antigens, for instance allergens, leads to IgG4 predominance. These antibodies continuously undergo half-antibody exchange and are thus functionally hetero-bispecific which implies that only one arm of the antibody binds to its cognate antigen and thus cannot effectively cross-link target antigens. IgG4 cannot activate complement because it is unable to bind the first complement cascade component C1q and also show little FcR binding. These two mechanisms are critical for the effector function of the IgG1-3 antibodies. Another clinically important feature of IgG4 antibodies is that their titers drop dramatically in response to RTX therapy, unlike the IgG1 antibodies (74, 75) indicating that IgG4 producing cells do not persist as long-lived plasma cells in contrast to many IgG1 producing cells. Accordingly, RTX has been proven useful in anti-Musk (IgG4) myasthenia gravis in a recent clinical trial (76), and it seems also to be effective in patients with CIDP and autoantibodies of the IgG4 isotype (see Treatment Implications).

Box 1 **Features of IgG4 antibodies.**

A sequential switching toward IgG3-IgG1-IgG2-IgG4 occurs with increasing levels of somatic hypermutations during a germinal center response.

Continuously undergo half-antibody exchange (functionally hetero-bispecific).

Cannot effectively cross-link target antigens.

Unable to bind C1q, so cannot activate complement.

Show little FcR binding.

Titers drop dramatically in response to RTX therapy.

The coexistence of IgG3 with IgG4 may contribute to the pathophysiology of anti-contactin-1 associated neuropathy by causing complement deposition, and this may be related to the IVIG-response of the patient (71). Further studies are necessary to understand the clinical importance of coexisting IgG subtypes.

## Epitope Mapping

Fn3-Fn4 region of the NF155 protein is unique to this neurofascin isoform and not found in the NF186 and NF140 isoforms. This specific region was found to be the main epitope based on the sera of two patients with anti-NF155 antibodies (27). This finding was confirmed in a larger study by using the FN1-4 region for epitope mapping. The authors found that 30/38 (79%) of the patients required Fn1-4 region to bind to the protein (28). Fn1-2 domains of NF186 interact with gliomedin and sodium channels, but the function of the Fn domains is not known for NF155 (77).

Neurofascin 155, NF186 and NF140 proteins are isoforms of the same protein. Whereas antibodies against NF155 recognize the Fn3-Fn4 domain that is unique to NF155, NF186/140 autoantibodies recognize the common Ig region (47). Indeed, sera from patients with NF186/140 antibodies reacted also to NF155 (47, 62). The authors concluded that these antibodies mainly react to NF186/140 *in vivo*, by showing that the sera of these patients mainly stain the nodes and AIS of in teased fiber preparations (47). It can be speculated that this is due to the sequestration of this domain when NF155 interacts with CNTN1 to form a complex. As expected, patients with high anti-NF155 titers found in this study did not react against anti-NF186/140 proteins.

For anti-CNTN1 antibodies, the main epitope region is the Ig domain which also plays a role in its binding to NF155. To be more specific, 8/10 sera bound to the Ig5-6 domain (56). Furthermore, glycosylation was found to be important for anti-NF155 antibodies to bind their targets (27), whereas anti-CNTN1 antibodies could bind both the glycosylated and deglycosylated proteins equally (56). Intriguingly, in one patient, reactivity to CNTN1 could only be detected when it was in complex with Caspr (45).

The main epitope region for anti-Caspr antibodies is not known yet. Autoantibodies against its homologous protein, Caspr2, bind mainly to the discoidin domain and do not require native protein structure (as they are still reactive when WB is used to test) or glycosylation (78).

## Pathogenicity of the Nodal Autoantibodies

A combination of humoral and cellular immunity is commonly assumed to contribute synergistically to the pathogenesis in CIDP (69). T cells break the blood-nerve barrier allowing access of serum proteins like antibodies to the nerve environment (69). The paranodal localization of NF155 suggests that disruption of the paranodal structure may be necessary before autoantibodies are able to bind *in vivo* (68, 69). Recently, neurofascin and compact myelin antigen-specific T cell response pattern have been analyzed in CIDP subtypes (79).

In Lewis rats immunized against peripheral myelin, early loss of NF186 and gliomedin and redistribution of potassium channels were found to precede demyelination, and this finding was associated with detection of antibodies against neurofascin and gliomedin (80). Transfer of pan-neurofascin mAbs (A12/18.1 mouse IgG2a and A4/4.3 mouse IgM) to Lewis rats in the beginning of clinical EAN caused enhancement and prolongation of the disease (27). In this model, the antibodies to NF enhanced a T cell-mediated pathology. In another study, nerve conduction studies showed that intraneural injection of A12/18.1 mAbs, induces reversible conduction block (81).

Recently, direct evidence on the pathogenesis patient-derived antibodies was obtained. First, it was shown that IgGs from patients with anti-CNTN1 antibodies prevent aggregation of Caspr/contactin1 cotransfected cells with the NF155 expressing cells in a cell aggregation assay (70). In the same study, patient IgGs caused nodal elongation and paranodal shortening in dorsal root ganglion/Schwann cell cultures. In another study (54), myelinated fibers of the skin from patients with anti-CNTN1 antibodies were analyzed by immunofluorescence. There was elongation of the nodes and loss of paranodal Caspr and/or neurofascin immunoreactivity providing the first morphological evidence of nodal/paranodal disturbance in seropositive CIDP patients (54). Importantly, axonal but not demyelinating neuropathy was detected in the sural nerves of these patients. Accordingly, demyelinating features were not prominent in the sural nerves of two patients in another study (52). Similarly, in a patient with anti-Caspr antibody positive CIDP, pathological diagnosis of the sural biopsy was axonal neuropathy and again there was severe dispersion of Caspr, neurofascin, and sodium channels in teased nerve fibers and dermal myelinated fibers (46). In these studies, T cell and macrophage infiltration were not reported to be prominent in the tissues examined.

IgG1 and IgG4 antibodies from CIDP patients with anti-CNTN1 IgG were purified and either applied by intraneural injections or incubated with isolated murine sciatic nerves (82). They found that IgG4 antibodies can pass through the PNJ slowly but progressively. In contrast, IgG1 and anti-Caspr2 IgG4 did not pass through the paranodal barrier. In the same study, passive transfer of IgG4 antibodies from patients with anti-CNTN1 IgG to Lewis rats after immunization with P2 peptide caused progressive clinical deterioration and ataxia and the pathological examination revealed selective loss of paranodal compartmentalization without any signs of axonal or demyelinating neuropathy. Finally, two studies investigated the sural nerves of anti-NF155 antibody positive CIDP patients ultrastructurally. In the first study, selective loss of transverse bands at the PNJ causing widening of the periaxonal space and infiltration of the Schwann cell processes were reported (83). The second study compared the histological and ultrastructural features of the sural nerves from 10 seropositive patients (9 with anti-NF155, 1 with anti-CNTN1) to 13 seronegative CIDP patients (60). Again, there was no obvious macrophage or cellular infiltration and demyelination in seropositive patients at the time of biopsy contrary to the seronegative group. There was a loss of transverse bands, detachment of the terminal myelin loops and widening of the periaxonal gap in around half of the paranodes examined. Teased-fiber examination showed a prominent axonal degeneration compared to antibody-negative CIDP patients and controls. There was a positive correlation between axo-glial detachment and axonal degeneration. Two recent studies showed that the level of anti-NF155 IgGs in serum is also related to functional parameters of the patients (84, 85). Electrophysiological measurements, including F-wave latency, distal latency motor conduction velocity, of three patients with CIDP and anti-NF155 antibodies changed in parallel with the serum autoantibody levels and also with grip strength test (84). Interestingly, in another patient, restoration of compound muscle action potential of the affected nerves, namely median and ulnar nerves, was observed after 18 years of disease onset and 14 years under steroid therapy (85). This observation suggests that a reversible conduction block similar to that in the acute motor axonal neuropathy (AMAN) form of GBS is possible also in seropositive CIDP.

How do the antibodies pass through the blood-nerve barrier? This barrier is permeable at the dorsal root ganglia, nerve roots, and end plate regions and antibodies may pass through these sites to travel to the nodes (5, 86). Of note, although sural nerve biopsies did not show any signs of prominent inflammation in the distal nerve, MR neurography studies showed prominent gadolinium enhancement and enlargement in nerve roots and CSF protein levels were found to be very high in seropositive CIDP patients in most studies, which may indicate the presence of inflammation and BNB disruption in the proximal nerves (52, 86).

Additional mechanisms yet unidentified are probably also in action. The contribution of coexisting complement-fixating IgG subtypes to pathogenesis was tested by *in vitro* studies. Binding of anti-CNTN1 auto-antibodies of three patients who had additionally IgG2 and IgG3 induced complement deposition and activation as measured by cell binding and ELISA-based assays (71). Whether this also happens *in vivo* is not known. In one study, sural nerve biopsy specimens from two patients (one positive for anti-NF155, the other for anti-CNTN1) were examined for complement deposition and both were negative (60). Furthermore, NF155 antibodies and the extracellular domain of NF155 inhibit myelination in myelinating cocultures (21). Thus, it is also likely that presence of these antibodies have a negative effect on remyelination after injury.

## The Concept of Nodopathy

Peripheral neuropathies are traditionally classified as either demyelinating or axonal. However, electrophysiological studies in patients with AMAN, a subtype of GBS, showed that these patients carry features that cannot be explained by any of these two categories (87). AMAN is associated with IgGs against GM1 gangliosides which were shown to disrupt the nodal structure without any overt demyelination in rabbits (88). These complementary clinical and preclinical findings led to the concept of nodopathy, which refers to a node/paranode based pathology, without any overt classical axonal or demyelinating features. Recently, this concept was widened to include neuropathies with different etiologies and autoantibodies that target the nodal and paranodal proteins (89). The disorders described in this review have many common clinical and laboratory features with the nodopathies, so they should not be regarded as demyelinating disorders, although they are clinically grouped under the chronic inflammatory “demyelinating” neuropathies at the moment. This point is worth to mention as it has treatment implications.

Seropositive CIDP differs from seronegative CIDP in terms of disease mechanism. In seronegative CIDP, the proximal nerve involvement is prominent and pathology shows segmental demyelination and other demyelinating features, T-cell and macrophage infiltration and a milder, secondary axonal degeneration (69). On the other side, in seropositive CIDP, there is absence of macrophage-mediated demyelination, inflammatory cell infiltration, and axonal pathology is more severe (52, 54, 60). The mechanism of axonal pathology in these disorders is not fully understood yet but is thought to result from the increased ion flux secondary to redistribution of sodium channels and disturbance of the metabolic, structural, and trophic support that comes from the glia (90).

This pathophysiological discrepancy is also reflected by the clinical phenotype of the patients (Table 6). Because of these differences, treatment and follow-up strategies should be tailored according to the autoantibody status of the patients, an implication which is supported by the studies reviewed in this paper. Depending on this recent shift in the CIDP paradigm, Kuwabara et al. stated that it may be better to term CIDP as a syndrome rather than a single homogenous disease (91). The significant differences in the clinical management of seropositive and seronegative CIDP patients from disease onset make autoantibody-testing in CIDP patients a necessity regardless of the rarity (~10%) of these antibodies.

**Table 6:** Summary of differences in the clinical phenotype between seropositive and seronegative chronic inflammatory demyelinating polyneuropathy (CIDP) patients.

	Seropositive CIDP				Seronegative CIDP
	Neurofascin 155	Contactin1	Caspr^a^	Neurofascin 186	
Age of onset, years	20–30	50–60	30	50–60	50–60
Subacute onset	++	++	++++	++++	+
Tremor	++	+	–	–	+
Sensory ataxia	+++	++++	–	++++	+
Severe pain	–	–	++++	–	Very rare
Central nervous system demyelination	+	–	–	–	Very rare
Intravenous immunoglobulin unresponsiveness	++++	+++	++++	++	++

*^a^Based on one CIDP case. Data presented in this table is mainly derived from Ref. (28, 46, 47). Frequencies were determined as follows: ++++ means between 80–100%; +++ means 50–79%; ++ means 20–49%; + means 5–19%; 5% > is very rare*.

## Treatment Implications

An important implication of testing antibody positivity in CIDP patients is the selection of the most suitable treatment approach for the individual patient. There are no prospective clinical trials yet; however, retrospective observations provided some insight regarding the treatment response (Table 7). IVIG treatment is not satisfactory in the majority of seropositive CIDP patients, especially in patients with anti-NF155 antibodies. In the study by Kadoya et al., 3/11 of the anti-NF-155 IgG patients responded to IVIG, whereas this ratio was 42/46 in the seronegative group (58). Similarly, the ratio of the patients with positive response to IVIG was less than 40% in other studies (28, 50, 52, 62). For patients with anti-CNTN1 antibodies, Miura et al. reported that 4/10 of the patients had a positive response to IVIG (56) and in two other studies, patients benefited from IVIG in the initial phase of the disease but only temporarily (45, 54). Similarly, one anti-Caspr IgG positive patient did not respond to IVIG. Poor response to IVIG may be explained by the lack of complement fixing capacity of the IgG4 subtype (71). Among patients with a poor response to IVIG, 6/8 patients responded favorably to corticosteroids or plasma exchange (58). Similarly, response to steroids or plasma exchange was more favorable in IVIG-resistant patients in other studies (28, 45, 52, 56, 62). In one study, four seropositive CIDP patients with resistance to corticosteroids and IVIG were treated with RTX (75). In all patients, antibody levels declined following treatment and three patients responded to therapy. In two of these patients, disease duration was less than 1 year and the treatment response was robust in these patients compared to the third patient with a long disease duration and limited RTX response (75). In another study, all three patients with anti-NF155 antibodies had a prominent response to RTX (62). Of note, one of these patients recovered from a locked-in state after RTX and cyclophosphamide. Additional singlet cases with seropositive CIDP and a robust response to RTX were also reported (46, 54). In a separate case with a devastating form of anti-NF155 antibody positive CIDP, who was resistant to all levels of treatments, autologous stem cell transplantation was tried and found to be very effective (92).

**Table 7:** Treatment response in seropositive chronic inflammatory demyelinating polyneuropathy patients.

Study	Steroid response	IVIG response	PE response	RTX response	Others	Reference
**Anti-NF155**						
Querol et al. (2014)	1/4 (partial)	0/4	2/2	None (*n* = 1)	CY none (*n* = 1)	(50)
Querol et al. (2015)	–	–	–	Good (*n* = 1) Partial (*n* = 1)	–	(75)
Ogata et al. (2015)	5/8	4/13	4/6	–	–	(52)
Kadoya et al. (2016)	Favorable	3/11	Favorable	–	–	(58)
Devaux et al. (2016)	15/29	5/25	Not good	–	–	(28)
Garg et al. (2017)	2/3	1/3	–	Good (*n* = 1)	MMF good (*n* = 1)	(61)
Burnor et al. (2018)	1/3	1/4	3/4	Good (*n* = 3)	CY good in 1/2 patients	(62)

**Anti-CNTN1**						
Querol et al. (2013)	3/3	2/3, only partial	–	–	CY no (*n* = 1), AZA partial (*n* = 1)	(45)
Querol et al. (2015)	–	–	–	Good (*n* = 1)	–	(75)
Miura et al. (2015)	8/11	4/10	–		CY no (*n* = 2)	(56)
Doppler et al. (2015a)	-	3/3 only at initial phase	1/1, only partial	Good (*n* = 1)	CY good (*n* = 1)	(54)

**Anti-Caspr**						
Doppler et al. (2016)	1/1, partial	0/1	1/1	Good (*n* = 1)	–	(46)

**Anti-NF186/140**						
Delmont et al. (2017)	3/5	3/4	1/2	Good (*n* = 1)	CY good (*n* = 1)	(47)
Burnor et al. (2018)	–	1/1 (temporary)	1/1 (temporary)	Good (*n* = 1)	CY favorable (*n* = 1)	(62)

*IVIG, intravenous immunoglobulin; PE, plasma exchange; RTX, rituximab; CY, cyclophosphamide; MMF, mycophenolate mofetil; AZA, azathioprine*.

Furthermore, studies suggest that it is especially important to implement a rapid and effective maintenance therapy regimen in seropositive patients after management of the initial relapse to prevent irreversible axonal degeneration. In the absence of maintenance therapy, progressive clinical decline is seen and irreversible axonal damage may pursue. Data on which therapies are effective for maintenance is scarce.

## Unanswered Questions

– Are any other yet unidentified autoantibodies implicated in CIDP or CCPD?
– High CSF protein and gadolinium enhancement of the nerve roots and plexuses indicate inflammation in proximal nerves which was not seen in sural nerve biopsies. What is the mechanism behind this?
– Why is seropositive-CIDP almost always associated with the IgG4 subtype whereas GBS patients have IgG1 or IgG3 subtype?
– How do subtle paranodal changes lead to severe axonal degeneration?
– What is the mechanism of tremor that is seen in patients with anti-NF155 antibodies? Do these antibodies pass to the CSF?
– Is nodopathy with or without the involvement of antibodies an important disease mechanism in CNS disorders?
– T cell response to nodal antigens in seropositive patients largely unexplored

## Conclusion

Discovery of the autoantibodies like anti-Aqp4 and anti-MOG in a fraction of patients with central demyelinating disorders changed our understanding and treatment of these disorders. Likewise, discovery of anti-neuronal antibodies in numerous CNS syndromes, recognition of their value as a biomarker and studies on the effect of these antibodies on CNS-pathology has dramatically contributed to the field of clinical neuroimmunology. Discovery of the autoantibodies against nodal antigens in some patients with CIDP has a potential to have a similar effect on the field of inflammatory neuropathies. Additional studies are necessary to elucidate the full spectrum of autoantibodies and their clinical characteristics.

## Author Contributions

EM conceptualized the article, contributed to writing, and made critical revision. AV contributed to design and writing of the article. KD made critical revision of the article.

## Conflict of Interest Statement

AV declares no conflict of interest. KD received a research grant from Kedrion and personal fees from Baxalta and Grifols. EM received honorarium from Roche, Novartis, and Genzyme and grant support from Novartis and Genzyme. He does not declare any specific disclosures related to this article.
